# Differences of Intra-Articular Graft Length between Sandwich-Style Reconstruction and Zhao-Style Non-Remnant-Preserving Double-Bundle Reconstruction of Posterior Cruciate Ligament

**DOI:** 10.1371/journal.pone.0155678

**Published:** 2016-05-16

**Authors:** Peng Shen, Xiaoxi Li, Caiqi Xu, Song Zhao, Shikui Dong, Yang Zhang, Jinzhong Zhao

**Affiliations:** 1 Department of Sports Medicine, Shanghai Jiao Tong University Affiliated Sixth People’s Hospital, Shanghai, China; 2 Department of Orthopaedics, Ruijin Hospital Affiliated to The Shanghai Jiao Tong University School of Medicine, Shanghai, China; 3 Department of Orthopaedics, Zhongda Hospital, School of Medicine, Southeast University, Nanjing, Jiangsu, China; Kanazawa University, JAPAN

## Abstract

Appropriate graft length within the joint and inside the osseous tunnel is essential for achieving posterior stability and adequate anchorage strength. Because of the curving path and thickness of the graft in double-bundle posterior cruciate ligament (PCL) reconstruction, especially in double-bundle PCL augmentation (with remnant preservation), the actual intra-articular length of PCL grafts, which remains unknown, may be longer than previously published values. The main purpose of the current study is to measure the actual intra-articular graft length required in sandwich-style PCL reconstruction (remnant-preserving double-bundle PCL augmentation) and Zhao-style non-remnant-preserving double-bundle PCL reconstruction (semi-anatomic double-bundle PCL reconstruction using double-double tunnel with tibial medial and lateral arrangement). Nine matched pairs of intact cadaveric knees were randomized between two groups and respectively received sandwich-style PCL reconstruction (remnant-preserving group) and Zhao-style non-remnant-preserving double-bundle PCL reconstruction (non-remnant-preserving group). The tunnel positions were exactly the same in two groups. The anterolateral (AL) bundle was reconstructed with four-stranded semitendinosus tendon, and the posteromedial (PM) bundle was reconstructed with four-stranded gracilis tendon. For each bundle, the length of the graft portion within the joint was measured. The current study indicated that in remnant-preserving group, the average intra-articular exposed portion was 42.0 mm (SD, 1.3 mm; range, 40.0 mm to 43.4 mm) for the AL bundle and 32.5 mm (SD, 2.9 mm; range, 27.8 mm to 35.8 mm) for the PM bundle. In non-remnant-preserving group, the intra-articular exposed portion was 34.5 mm (SD, 1.0 mm; range, 32.7 mm to 36.0 mm) for the AL bundle and 29.1 mm (SD, 2.1 mm; range, 25.2 mm to 31.9 mm) for the PM bundle. For both the AL and PM bundles, significant differences were found in average intra-articular graft length between the two groups. The current study, whose methodology is more rigorous and accurate by measuring the actual intra-articular graft length, has direct applications to clinical practice. When considering the total graft lengths during reconstruction, it is necessary to recognize that remnant PCL has a space occupation effect on graft and that remnant preservation requires longer intra-articular graft lengths than non-remnant preservation.

## Introduction

Certain factors implicated in the failure of posterior cruciate ligament (PCL) reconstruction are similar to those identified in ACL failure, such as graft selection, graft size, tunnel placement, fixation method, rehabilitation.[1–6] The strength of PCL grafts has a notable effect on graft in situ force and joint posterior stability, [3] making graft thickness crucial for satisfactory PCL reconstruction outcomes. Clinically, surgeons fold the original tendon graft as many times as possible to increase graft thickness and strength, which reduces the eventual graft length as the number of folds increases. However, sufficient intra-articular graft length can significantly restore kinematic function and posterior stability.[7,5] Concurrently, adequate fixation and tendon-to-bone healing may require sufficient intra-osseous graft length.[8–11] Striking the right balance between increasing graft thickness and maintaining sufficient graft length is essential in graft preparation. As a result, precise information regarding appropriate intra-articular and intra-osseous graft length for PCL reconstruction should be determined.

Work by Girgis et al.[8] suggested that the average length of the PCL was 38.0 mm. In their study, the PCL was intact, and the entire length of the ligament was measured in situ, including its broad, sweeping attachments to the tibia and femur. Similarly, Miller et al.[7] reported the intra-articular PCL graft length by measuring the centre-to-centre distance between the insertion sites of the PCL graft, which suggested that the average PCL graft intra-articular distance was 30.7 mm (standard deviation, 2.6 mm), with a range of 28.0 to 36.0 mm. However, the graft exhibits curvature at the orifices and along its path in the actual situation. The curvature of the graft reportedly increases intra-articular graft length, [9] making it important to recognize that the actual intra-articular PCL graft length differs from previously PCL lengths or the centre-to-centre distance of the insertion sites for PCL grafts.[7,8]

Double-bundle PCL augmentation (with remnant preservation), also called sandwich-style PCL reconstruction, is one option for the treatment of PCL-deficient knees, and satisfactory outcomes have been reported.[10] Clinically, Zhao-style non-remnant-preserving double-bundle PCL reconstruction (semi-anatomic double-bundle PCL reconstruction using double-double tunnel with tibial medial and lateral arrangement), which has the same tunnel positions as the sandwich-style PCL reconstruction does, has been another option for years and the International Knee Documentation Committee (IKDC) rating scale were graded as normal or nearly normal in about 93% of the patients at more than 2 years’ follow-up (Jinzhong Zhao, unpublished data, 2006). Graft thickness and path curvature must be taken into account in these techniques when determining the appropriate intra-articular graft length. Particularly in clinical scenarios of sandwich-style PCL augmentation, the remnant PCL fibres partly cover around the graft near the tunnel aperture and exist between two bundles at their middle substantial part. As a result, the preserved remnant fibres inevitably occupy spaces, and this space occupation effect of remnant fibres on the intra-articular portion of the graft should not be ignored, which is the main reason for the curvature of the graft. A more curved graft path will result in a longer intra-articular graft, as previously reported.[9] Consequently, previously published graft lengths may lead to the inaccurate use of tendon strands in clinical practice, resulting in less tissue in the osseous tunnel, which could possibly affect the intra-osseous anchoring strength and ingrowth of the graft.[11–13] Thus, it is critical for us to determine the actual intra-articular graft length with rigorous methodology.

As the required graft length can be determined via double-bundle PCL reconstruction in cadavers, the purpose of current study was to measure the actual intra-articular graft length for sandwich-style PCL reconstruction and Zhao-style non-remnant-preserving double-bundle PCL reconstruction. Our hypothesis was that longer grafts, for both AL- and PM-bundle reconstruction, were needed in double-bundle PCL augmentationthan Zhao-style non-remnant-preserving double-bundle PCL reconstruction, which was mainly due to the curvature of the graft caused by the space occupation effect of the remnant PCL.

## Materials and Methods

The cadaveric specimen in current study were obtained from The Red Cross Society of China Affiliated Shanghai Fudan University Body Donation Station (http://www.redcross-sha.org/view.aspx?id=5199), which has been cooperating with Department of Sports Medicine, Shanghai Jiao Tong University Affiliated Sixth People’s Hospital since 2013. The sources of specimen are legal and the informed consent were obtained by The Red Cross Society of China Affiliated Shanghai Fudan University Body Donation Station.

### Specimen preparation

A total of 9 match-paired knees from fresh-frozen human cadavers of varying size and sex (mean age, 64 years; range, 52 to 72 years; 5 males and 4 females; 9 right and 9 left) were used in this study. Knees in each bilateral pair were randomly assigned to either of two groups: double-bundle PCL augmentation (with remnant preservation) or Zhao-style double-bundle PCL reconstruction without remnant preservation. All of the knees were macroscopically intact and demonstrated no evidence of previous surgery, injury, disease or tissue abnormality that may affect normal knee function. All specimens were maintained at -20°C and thawed at room temperature for 24 h before the experiment. Then, dissections consisting of stripping away the skin, superficial fascia, muscles and synovial sheath without disturbing the soft tissues covering the posterior femoral condyle and all the ligaments around knee were performed carefully to clearly expose the PCL and delineate its surface fibre pattern and osseous insertion sites, with the essential stability of the knee maintained. After preparation, the specimen was mounted in the stabilizing rig.

### Graft harvesting and preparation

Both the semitendinosus and gracilis tendons were harvested in a routine manner.[14,15] Both ends of the tendons were sutured with No. 2 polyethylene sutures. Each tendon was folded twice to make a four-strand graft. After pre-tensioning under a force of 80 N for at least 5 min to minimize creep effects, the length and thickness of the graft were measured.[10] To simulate the common condition of grafts in clinical practice and to ensure that no bias existed in the selection of the grafts between the two groups, extra cadaveric tendon strands were prepared and specific selection criteria were applied: Only four-strand semitendinosus tendons with a diameter of 8.0 mm at the proximal end were used to reconstruct the anterolateral (AL) bundle, whereas the diameter of the distal end was 0.5 mm larger. Meanwhile, only four-strand gracilis tendons with a diameter of 6.0 mm at the proximal end were used to reconstruct the posteromedial (PM) bundle, whereas the diameter of the distal end was 0.5 mm larger.

### Surgical procedure

**Fig 1:**
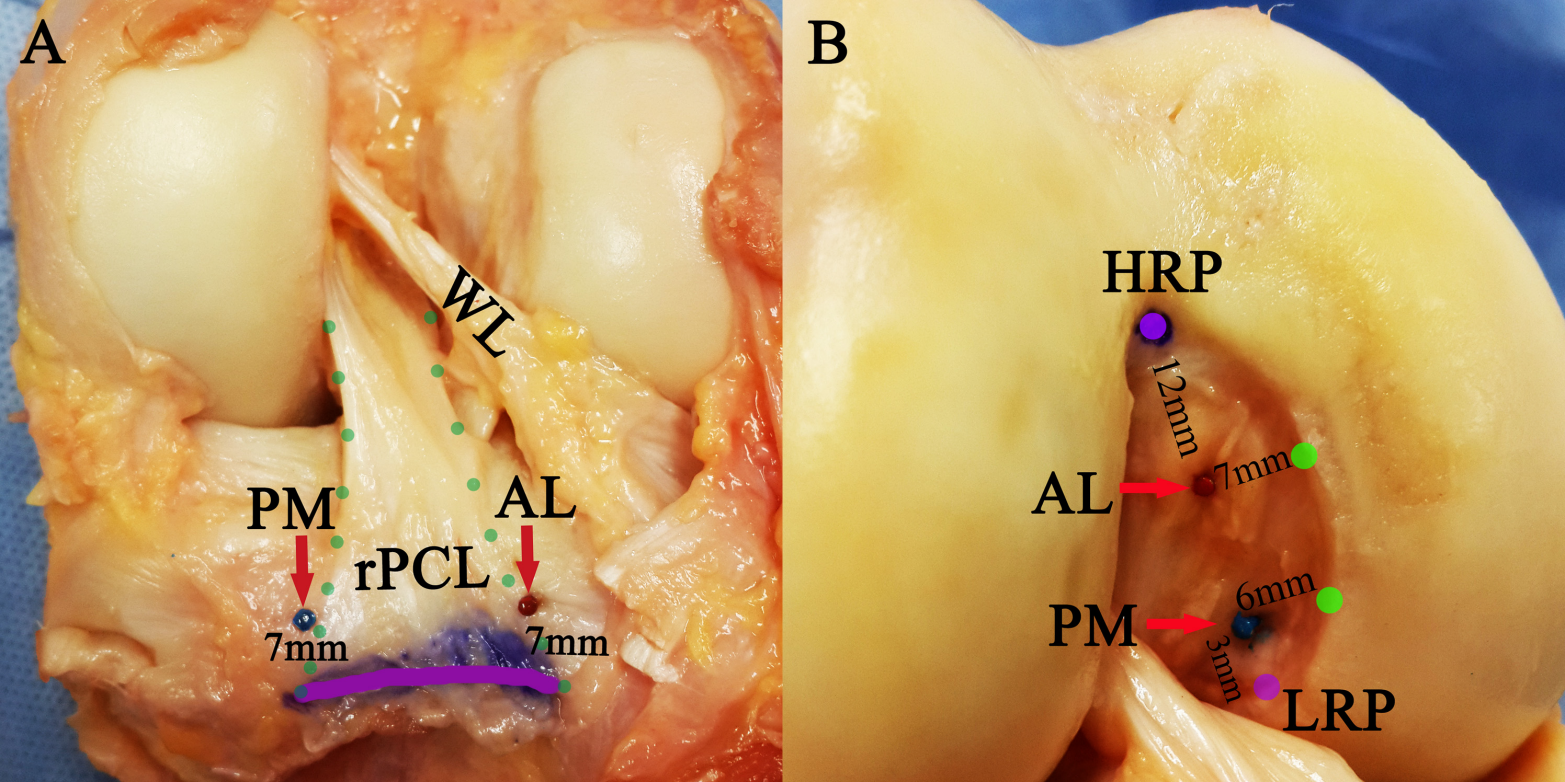
Locations of the tibial and femoral tunnel. (A) Tibial tunnel sites. The green points outlining the remnant of posterior cruciate ligament (PCL) and the purple line marking the most posterior edge of the PCL footprint (AL, the center point of the tibial tunnel of the anterolateral bundle graft; PM, the center point of the tibial tunnel of the posteromedial bundle graft; WL, Wrisberg ligament; rPCL, the remnant of PCL). (B) Femoral tunnel sites. The reference sites at distal cartilage edge marked with green points. (AL, the center point of the femoral tunnel of anterolateral bundle graft; PM, the center point of the femoral tunnel of posteromedial bundle graft; HRP, high reference point; LRP, low reference point)

Sandwich-style PCL reconstructions and Zhao-style non-remnant-preserving double-bundle PCL reconstructions were performed respectively, with the same locations of the inner orifices of the tibial and femoral tunnels. Locations of tunnel apertures in this study were based on our clinical practice and were exactly the same as those used in our current clinical procedures, of which relatively satisfactory clinical outcomes had been reported.[10] The two tibial tunnels were centred 7.0 mm anterior to the most posterior edge of the footprint in the lateral and medial part of the footprint (Fig 1), with the diameter corresponding to that of the graft distal end. The femoral tunnel for the AL bundle was centred 12.0 mm posterior to the midpoint of the anterior edge of the intercondylar notch roof (a reference landmark described as a high reference point or HRP) and 7.0 mm proximal to the distal cartilage edge and that for the PM bundle was located 3.0 mm anterior to the most posterior edge of the footprint (described as a low reference point or LRP) and 6.0 mm proximal to the distal cartilage edge (Fig 1). Both femoral tunnels consisted of an inner portion with a diameter equal to the graft proximal end and a length of 25.0 mm, as well as an outer portion, with a diameter of 4.5 mm, that extended to the femoral outer orifice.

In the remnant-preserving group, the original PCL thickness was measured and approximately 20% of the fibres from the substantial part of the PCL were stripped off using a scalpel to simulate the conditions of the PCL remnants that we observed in clinical practice. Then two reconstructed PCL bundles were inserted, passing through and along the remnant PCL fibres with the curvature at the inner orifices and along the path which was caused by the space occupation of remnant PCL, whereas in the non-remnant-preserving group, the original PCL fibres were completely removed, leaving the two newly reconstructed bundles connecting the femur and tibia, with the curvature only at the inner orifices.

After passing through the joint and two tunnels, the grafts were fixed using the suspension fixation, with the AL bundle fixed at 90 degrees of knee flexion and the PM bundle fixed at full knee extension. The proximal end of the graft was first fixed. Before the final distal graft fixation, the knee was manually moved through its full range of motion 5 times to ensure a proper passage of the grafts, to remove any slippage in the tunnels and to obtain a natural intra-articular state for the grafts. Then with an approximately 80-N traction force applied to the graft at the distal end in line with the tunnel using a graft tensioning device (Arthrex Inc.) as well as the anterior tibial force imposed to simulate the anterior drawer which maintained the knee at appropriate anatomical position, the distal end of the graft was fixed.[16–18]

### Measurement

**Fig 2:**
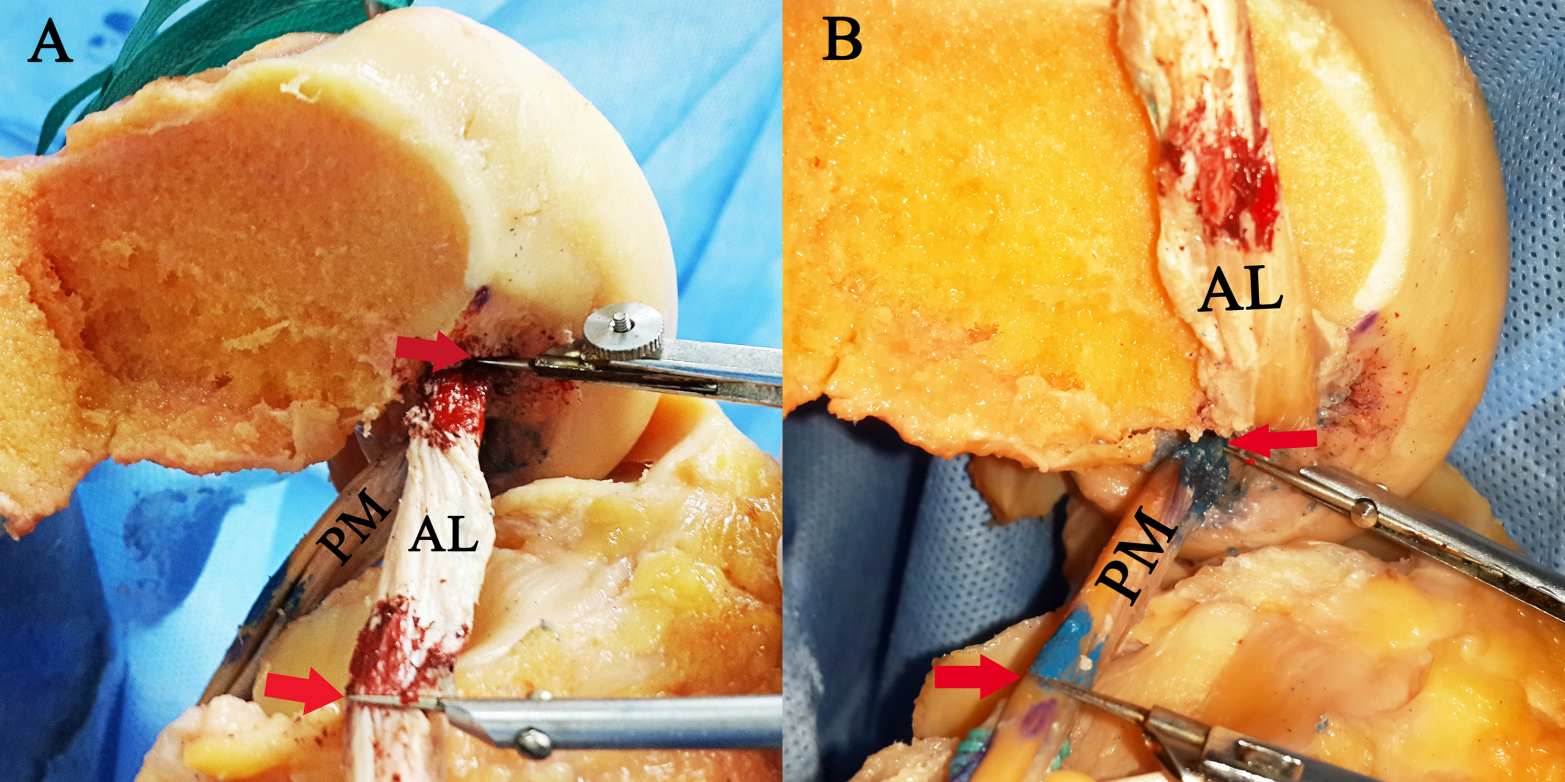
Measurements of the intra-articular graft length. (A) The measurement of the intra-articular length of anterolateral bundle graft. (B) The measurement of the intra-articular length of posteromedial bundle graft. (AL, anterolateral bundle graft; PM, posteromedial bundle graft; Red arrows, edge of the intra-articular portion of the graft)

**Fig 3:**
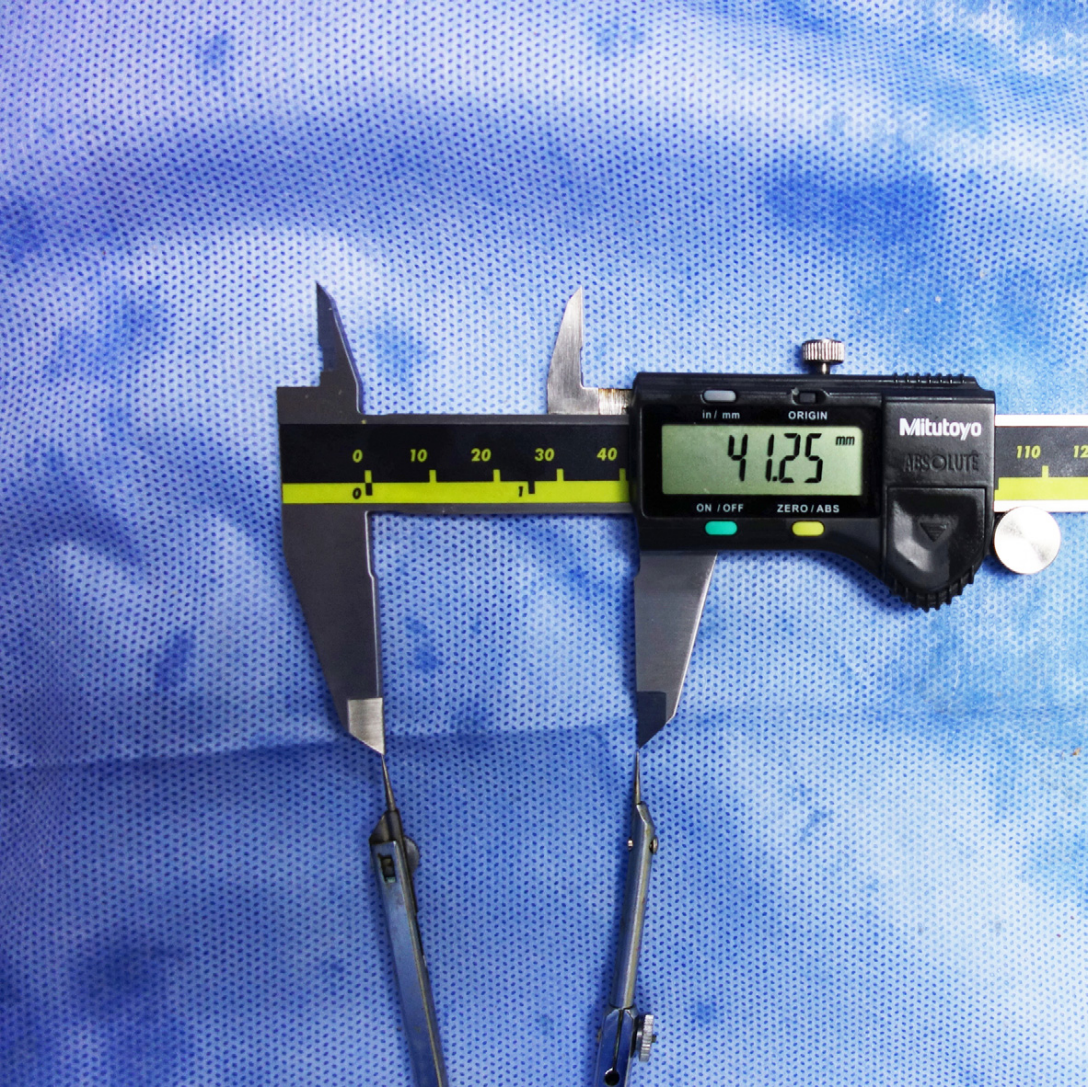
The compass and digital calliper with an accuracy of 0.01 mm used in measurements.

The grafts were marked at the inner orifices of the four tunnels. The distal ends of the grafts were released from fixation and pulled out of the tibial tunnels. The lateral femoral condyle and native PCL fibres were removed before the measurements. With suitable tension on the graft in line with the intra-articular segment, the longest distance between the marked lines on the graft were measured using a compass and a digital calliper (Mitutoyo, Japan) with an accuracy of 0.01 mm. (Figs 2 and 3). To decrease inter- and intra-observer variability, each distance was measured twice by two independent, blinded observers.

### Statistical analysis

Power analysis was first performed using PASS 11 software (version 11.0.7, NCSS, LLC; Kaysville, Utah, USA) according to a pilot study of 4 cadaveric knee pairs and related literature, [19,9,7] which indicated that a minimum sample size of nine knees per group was necessary for an error probability of 0.05 and a power of 0.8. All data were first confirmed to be normally distributed using the Shapiro-Wilk test. The equality of variance was checked using the Levene test. A 2-sample independent t-test was performed to compare differences in the average intra-articular graft lengths of certain bundles between the remnant-preserving and the non-remnant-preserving groups. To evaluate the reliability of the measurements, intra- and inter-observer variability were assessed by calculating the intra-class correlation coefficient (ICC), which was interpreted as poor if less than 0.4, marginal if equal to or greater than 0.4 but less than 0.75, and good when equal to or greater than 0.75. Statistical significance was set at p<0.05. All statistical analyses were performed using SPSS software (version 12.0, SPSS, Chicago, IL, USA).

## Results

The intra-articular graft lengths of all specimens are summarized in Table 1. In double-bundle PCL augmentation (remnant-preserving group), the mean intra-articular graft length was 42.0 mm (standard deviation, 1.3 mm) for the AL bundle, with a range of 39.5 to 43.4 mm and a 95% confidence interval (CI) of 41.1 to 43.0 mm, and was 32.5 mm (standard deviation, 2.9 mm) for the PM bundle, with a range of 27.8 to 35.8 mm and a 95% CI of 30.4 to 34.7 mm. For Zhao-style double-bundle PCL reconstruction (non-remnant-preserving group), the average intra-articular graft length for the AL bundle was 34.5 mm (standard deviation, 1.0 mm), with a range of 32.7 to 36.0 mm and a 95% CI of 33.7 to 35.3 mm, and for the PM bundle, was 29.1 mm (standard deviation, 2.1 mm), with a range of 25.2 to 31.9 mm and a 95% CI of 27.5 to 30.7 mm.

**Table 1:** Intra-articular graft lengths. The values are the mean data of the measurements conducted by two independent blinded observers. (AL, anterolateral; PM, posteromedial; R, right knee; L, left knee).

Remnant-preserving group			Non-remnant-preserving group		
Specimen No.	AL bundle (mm)	PM bundle (mm)	Specimen No.	AL bundle (mm)	PM bundle (mm)
1R	42.46	33.55	1L	34.87	29.50
2R	39.50	27.75	2L	32.74	25.23
3L	43.27	35.76	3R	35.81	31.31
4R	41.65	31.98	4L	34.50	28.76
5L	40.69	29.90	5R	33.69	26.68
6L	43.43	35.43	6R	36.03	31.88
7R	42.14	32.65	7L	34.67	29.06
8L	42.23	35.68	8R	33.89	30.02
9L	42.99	30.21	9R	34.24	29.24

**Fig 4:**
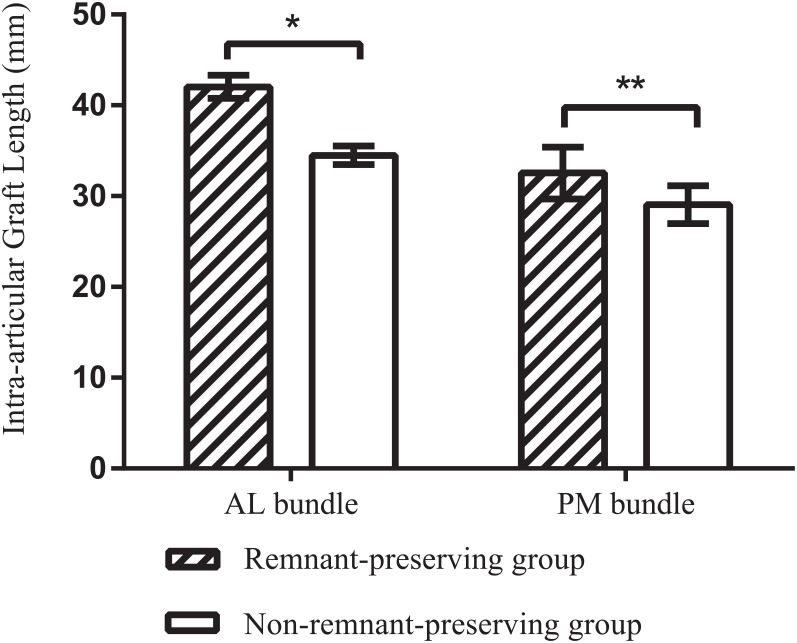
Comparison of average intra-articular graft lengths, presented as mean ± SD. (AL bundle, anterolateral bundle; PM bundle, posteromedial bundle). *- Two-sample independent t test for the comparison of the intra-articular length of AL bundle graft between two groups, t value is 13.844, p<0.001. **- Two-sample independent t test for the comparison of the intra-articular length of PM bundle graft between two groups, t value is 2.949, p<0.01.

When comparing the intra-articular graft lengths between the remnant-preserving and the non-remnant-preserving groups, significant differences were found for both the AL (p<0.001) and PM bundles (p<0.01). For both bundles, the remnant-preserving grouphad a longer intra-articular graft length than non-remnant-preserving group(Fig 4).

Both the intra- and inter-observer reliability of all measurements were high according to the ICC, which was greater than 0.9 for intra-observer reliability, ranging from 0.92 to 0.99, and was greater than 0.8 for inter-observer reliability, ranging from 0.89 to 0.96.

## Discussion

In the current paper, we measured intra-articular graft length for sandwich-style PCL reconstruction (remnant-preserving double-bundle PCL augmentation) and Zhao-style non-remnant-preserving double-bundle PCL reconstruction (semi-anatomic double-bundle PCL reconstruction using double-double tunnel with tibial medial and lateral arrangement) and observed significant differences upon comparison of the average intra-articular graft length between two groups. What’s more, we also provided a method more accurate for measuring the real intra-articular graft length, which can be used in future studies with regard to the appropriate intra-articular graft length in different surgical methods.

Our results indicated that the minimum intra-articular graft length for AL bundle reconstruction should be 39.5 mm in sandwich-style technique and 32.7 mm in Zhao-style technique. As for PM bundle reconstruction, the minimum intra-articular graft lengths were 27.8 mm and 25.2 mm in sandwich-style technique and Zhao-style technique, respectively. Our results differed from those of Miller et al., [7] who reported a PCL graft intra-articular distance of 30.7 mm (standard deviation, 2.6 mm), with a range of 28.0 to 36.0 mm. These differences were undoubtedly caused by the detachment of the PCL, the tunnel sites for single-bundle PCL reconstruction and the use of centre-to-centre distance measurements in their study. Jung et al.[9] demonstrated that graft curvature increases the intra-articular graft length. Moreover, the thicker the graft, the larger the likely curvature at the orifices. The centre-to-centre distance was shorter than the actual intra-articular graft length because graft thickness and curvature were ignored. Therefore, by simulating PCL reconstruction using real tendon grafts to better reproduce the clinical conditions of PCL grafts within the joint and by measuring the actual length of the graft portion within the joint, we are able to account for graft curvature and present a more precise value for the intra-articular graft length.

Our results also support the hypothesis that longer grafts, for both AL- and PM-bundle reconstruction, are needed in sandwich-style double-bundle PCL augmentation (with remnant preservation) than Zhao-style non-remnant-preserving double-bundle PCL reconstruction, which mainly results from the curvature of the graft caused by the space occupation effect of the remnant PCL fibres. As a consequence of this space occupation effect of remnant PCL, the fact that the difference in the intra-articular graft length of the AL bundle between the two groups is larger than that for the PM bundle may be because the AL bundle graft path, which passes over the remnant, is more curved than that of the PM bundle graft, which passes directly under the remnant, as reported by Jung et al.[9] The same may be true for single-bundle PCL reconstruction with and without remnant preservation, which must be confirmed in the future.

Our results have direct applications to our clinical practice. When considering the total graft lengths during reconstruction, it is necessary to recognize that technique with remnant preservation requires longer intra-articular graft lengths than non-remnant-preserving techniquebecause of the space occupation effect of the PCL remnants. According to recent studies, [11,12] sparing a 20-mm intra-osseous graft length would be a relatively safe practice. For instance, based on values reported by Miller et al.[7] an original tendon with a minimum total length of 280.0 mm is sufficient for preparing a 4-stand graft with a length of 70.0 mm (30.0 mm within the joint, 20.0 mm in each osseous tunnel). However, according to our study, in sandwich-style PCL reconstruction, the graft length for the AL bundle after folding should be at least 79.5 mm (39.5 mm within the joint, 20.0 mm in each osseous tunnel), which is 9.5 mm longer than the 70.0 mm we previously believed. As a consequence, if we were to prepare a 4-strand graft to maintain sufficient strength, the total length of the original tendon would be 318.0 mm. This requirement is not easily met, especially when using autografts, where sufficient graft thickness and length are difficult to achieve simultaneously due to individual differences and source limitations. Therefore, good graft preparation must balance increasing graft thickness and maintaining sufficient graft length. A 6-strand graft made of two 3-strand grafts may be a good choice to simultaneously achieve sufficient strength and length in this situation because it only requires the original tendon to have a total length of 238.5 mm. Moreover, our study also suggests that 67.8 mm (27.8 mm within the joint, 20.0 mm in each osseous tunnel) is the minimum graft length for reconstructing the PM bundle in sandwich-style PCL reconstruction. As for Zhao-style non-remnant-preserving double-bundle PCL reconstruction, the minimum graft length is suggested to be 72.7 mm (32.7 mm within the joint, 20.0 mm in each osseous tunnel) for the AL bundle and 65.2 mm (25.2 mm within the joint, 20.0 mm in each osseous tunnel) for the PM bundle.

However, this study still has certain limitations. First, it is hard for us to simulate the natural state of the PCL remnants on cadaveric knees. Even though the diameter of remnant PCL differs in different patients, it is necessary for us to set a standard of the residual quantity to make the results in this study comparable. Therefore, based on our clinical observation, 80% of the PCL was preserved to simulate the PCL remnants. In theory based on our results, less remnant fibres lead to shorter graft length, which should be considered when applying our findings in a clinical scenario. Secondly, the difference between the open operation in our current study and the arthroscopic operation in clinical practice may cause systematic errors, which is inevitable. The lack of knee kinematic or laxity examination after the PCL reconstruction is another limitation, but fortunately the fact that we had maintained the knee at appropriate anatomical position during the final distal graft fixation was helpful to verify our procedures.

## Conclusion

Sandwich-style PCL reconstruction (remnant-preserving double-bundle PCL augmentation) required a longer graft within the joint than Zhao-style non-remnant-preserving double-bundle PCL reconstruction (semi-anatomic double-bundle PCL reconstruction using double-double tunnel with tibial medial and lateral arrangement). For sandwich-style technique, the minimum intra-articular graft lengths were 39.5 mm and 27.8 mm for AL bundle and PM bundle, respectively. As for Zhao-style technique, the minimum intra-articular graft lengths were 32.7 mm and 25.2 mm for AL bundle and PM bundle, respectively. As a consequence of the space occupation effect of the remnant PCL, with similar osseous tunnel length, the total graft length required is longer in the technique with remnant preservationthan in that without remnant preservation.
